# Determining the optical properties of solar cells using low cost scanners

**DOI:** 10.1038/s41598-022-21229-w

**Published:** 2022-10-21

**Authors:** Mattias Klaus Juhl, Binesh Puthen Veettil, Giuseppe Scardera, David Neil Roger Payne

**Affiliations:** 1 Macquarie University, Sydney, NSW 2113 Australia; 2 University of New South Wales, Kensington, NSW 2052 Australia

**Keywords:** Solar energy, Imaging and sensing

## Abstract

This paper investigates the use of consumer flatbed scanners for the use of monitoring solar cell precursors. Two types of scanners are investigated a contact image scanner and scanners with more conventional optical setups. The contact image sensor is found to be more suitable as it does not require additional flat field calibration. The scanners’ ability to monitor variation in sample texture was investigated by monitoring the reflection of multi-crystalline and mono-crystalline textured wafers. For a baseline, a comparison was made to a high-end tool used in industry. Both good qualitative agreement and statistical correlation were achieved between the scanner and industry tool for the isotropic multi-crystalline wafers.

## Introduction

The consumer electronics market has resulted in technologically complex equipment designed for specific everyday uses being available off the shelf at a very low-cost (<$100 USD). If such items can be leveraged for unintended niche applications, they can substantially lower equipment costs, lead times, and open up access for more people, e.g. researchers on limited budgets. This paper investigates the conventional office-use flatbed scanners for optical characterisation of solar cells. Flatbed office scanners are widely available, have typical costs in the hundreds of dollars, provide high spatial resolution (40 $$\upmu $$m pixel resolution on low cost scanners), and include a built-in white light source enabling measurements across three different colours channels. Office Scanners have found similar unintended uses for monitoring radiation^1–4^, a range of biomedical imaging applications^5^ and for monitoring components’ physical dimensions^6^. The same types of scanners looked at in this paper have also previously been analysed for use in holographic microscopy^7^.

High efficiency, low cost solar cells’ performance depends on their electronic and optical properties. As solar cells are large area devices (greater than 220 cm$^2$ wafers), it can be challenging to maintain uniform optical properties. To monitor these properties, specifically the reflection, solar cells are often measured with stand alone point by point mapping tools, such as tools from Semilab^8^ or the LOANA from pv-tools as used in this paper. Pointwise spectral mapping of solar cells can take more than 20 min per sample for millimetre resolution. With production speeds of over one sample per second per line, this is much too slow to monitor every sample in manufacturing. Such tools also cost several hundred thousand dollars, and are out of reach for many research groups. The use of flatbed scanners may address both of these disadvantages.

In this paper, we demonstrate the optical characterisation of solar cells using a consumer-grade flatbed scanner. We first compare the two common types of flatbed scanner technology widely available, showing significantly different results. Based on the analysis of these results a preferred type of scanner is selected and is then experimentally investigated as a means to monitor the variation of texturing across a silicon solar cell wafer substrate. We compare the results from the scanner, obtained in less than 10 seconds to that of a high quality laboratory based tool that takes 20 min.

## Results

First, measurements on a textured silicon wafer with a contact image sensor (CIS) and non-CIS based flatbed scanner are presented. Significant differences between them are observed, with artifacts present in the non-CIS scanner data. Following on from this, the CIS scanner is used as it does not suffer from this effect. The CIS scanner is then used to examine a variety of textured mono-crystalline and multi-crystalline wafers. Scans are measured and compared to reflection maps obtained on a high end laboratory tool.

### Comparison of scanner types

**Figure 1:**
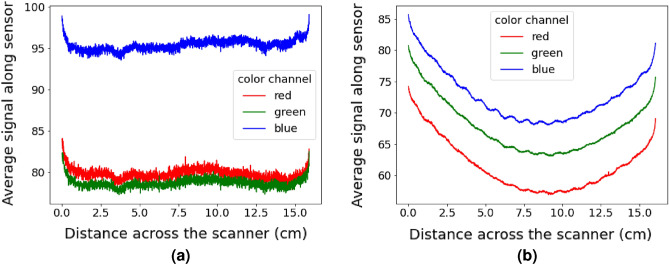
Comparison of the signal from the blue channel averaged across the wafer in the scanners direction of movement. Each dot represents the average signal measured by a different pixel for (**a**) a CIS and (**b**) non-CIS scanner.

To compare the CIS and non-CIS scanners, scans were taken of the same wafers with both scanners. The first wafer imaged was a p-type random pyramid textured mono-crystalline wafer. There was a clear difference between the scans across the axis of imaging. To highlight this, line scans representing an average over the wafer along the axis of motion are shown in Fig. 1. The axis of motion is described in the “Methods” section and labelled in Fig. 6. The CIS sensor measured a relatively constant signal across the sample while the non-CIS scanner measured a large bow and a small wavy feature across the sample. These features remained on this imaging axis if the sample was rotated, suggesting that the bow and ripple are likely the results of differences between the scanners’ imaging optics.

**Figure 2:**
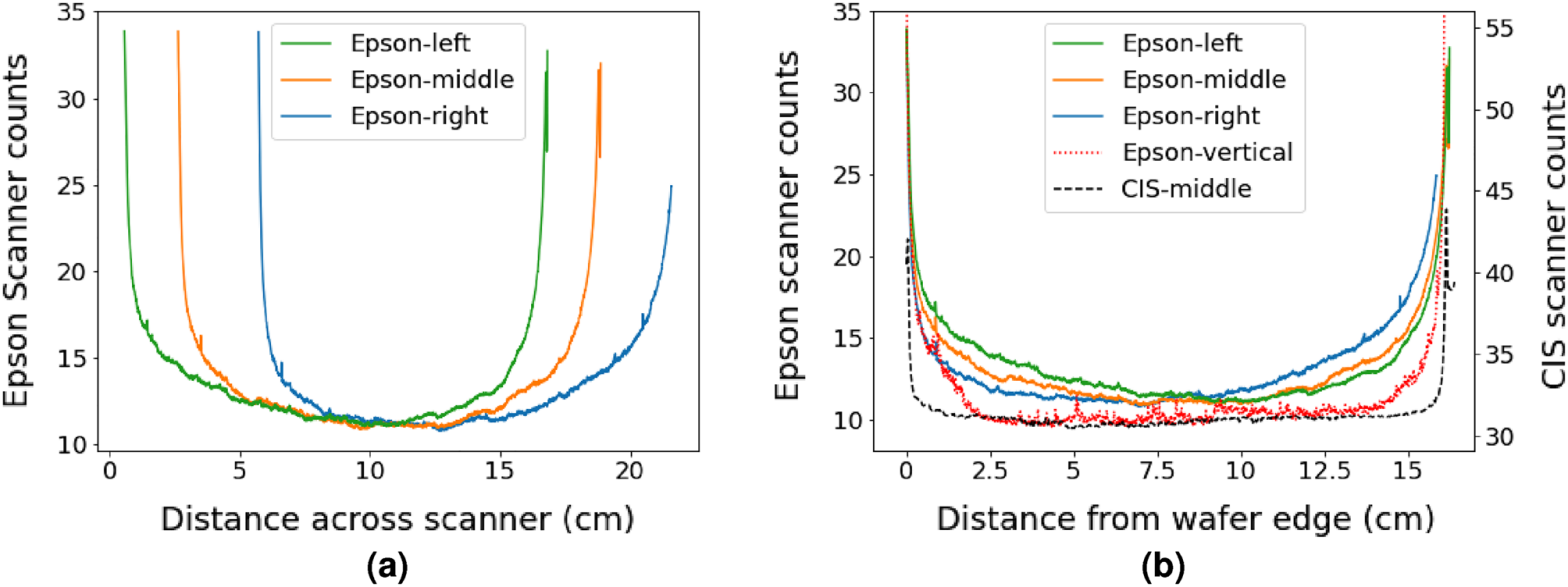
Repeat measurement of the same sample demonstrating the increase in the measured signal at regions away from the center of the scanner head for a CCD based Epson V800 scanner. Note the Epson V800 has a larger imaging axis compared to the Canon Lide 300, as was used to produce Fig. 6.

To further investigate this effect, additional measurements were performed on a textured p-type mono-crystalline wafer coated with SiN_x_, a material that is used as an anti reflection coating for solar cells. Three measurements were taken of this wafer with the non-CIS scanner (Epson V800). After each measurement, the wafer was translated to the right across the scanner’s imaging axis. The same line scans as in Fig. 1 were extracted from the green channel and are shown in Fig. 2a. A difference in the measured bow occurs between the three measurement locations. The bow is more easily observed when re-plotting the data as a function of distance from the edge of the wafer, as is shown in Fig. 2b. For parts of the wafer close to the centre of the scanner the counts are lower, and these counts increase as the wafer moves to the edge of the scanner. This is most clearly seen in the measured intensity of the left side of the wafer decreasing as the wafer is moved away from the left edge of the scanner. Equivalent effects are observed on the right side of the wafer when it is located further away from the right side of the scanner. Also shown in Fig. 2b is data averaged along the imaging axis rather than the axis of motion and is labelled as Epson-vertical, and the same wafer imaged with the CIS based Canon LiDe 300 is labelled as CIS-middle. The Epson-vertical data was taken from the same image as Epson-middle. These two additional lines are notably flatter than the original data. This highlights that the wafer bow is a systematic response from the Epson V800, occurring strongly along the axis of imaging, and not related to the sample’s reflection. Again, when the sample was rotated and re-measured the bow remained on the imaging axis.

### Monitoring solar cell texturing

**Figure 3:**
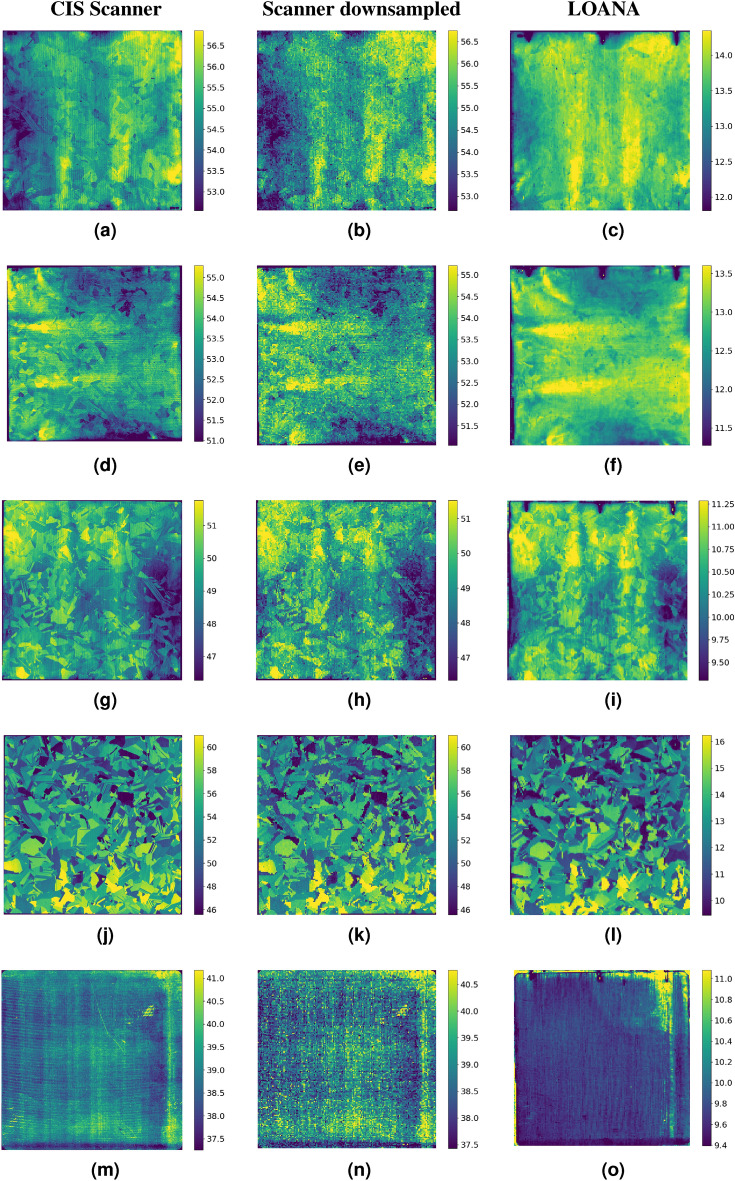
Comparison of the blue channel of scans on a CIS based scanner of four multi-crystalline wafers (**a**–**i**) and one mono-crystalline wafer (**m**–**o**) taken with the CIS scanner and a hemispherical reflection map at 405 nm taken with the LOANA. The images going from left to right in each row are acquired with the scanner, the scanner but downsampled to the resolution measured by the LOANA, and finally, the LOANA. The samples shown in this figure are in each row, with the first row showing Multi-1, followed by Multi-2, Multi-3, Multi-4 and finally Mono.

As the CIS based flatbed scanner was not affected by the artifacts that impact the non-CIS scanner, only the CIS scanner was used to monitor texturing of solar cell texturing. A CIS based flatbed scanner was used to monitor the spatial variation in the texture of multi-crystalline and mono-crystalline wafers prepared with varying texture conditions. To validate that the scanner can be used to monitor changes in sample reflection, measurements were also taken using a LOANA (PV-tools). The results of these measurements are shown in Fig. 3, with a different wafer shown in each row. The first four rows are metal assisted chemically etched multi-crystalline wafers, each etched using a different recipe, while the last row is an anisotropic alkaline etched mono-crystalline wafer. The first column of Fig. 3 shows the image taken from the scanner at a resolution of 118 pixels per centimetre, being a pixel pitch of approximately 85 um. The second column shows a down-sampled version of the scanned image to match the lower resolution of the image taken by the LOANA. The final column shows the measurement taken by the LOANA, with a pixel pitch of 1 mm.

**Figure 4:**
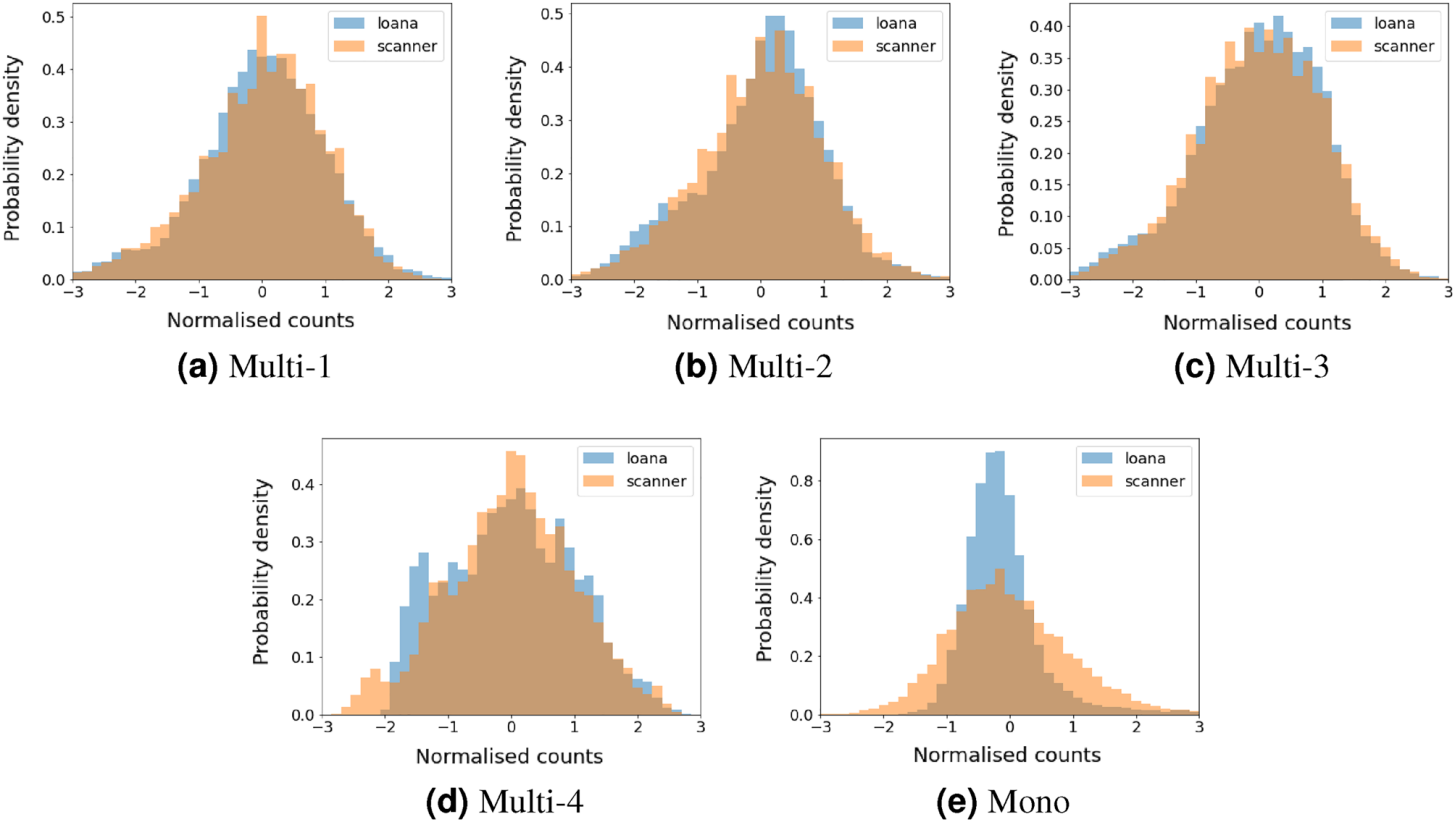
Normalised frequency histograms of the signal received on the samples from both the LOANA and CIS based scanner. Samples are shown in the same order as Fig. 3.

To quantify the similarity between the measurements several methods were used. The first method compares the frequency distribution of a normalised count rate, as shown in Fig. 4. The data was first normalised by subtracting the mean and then dividing by the standard deviation. Good agreement is observed for all samples, with both the scanner and LOANA measuring similarly shaped distributions.

**Figure 5:**
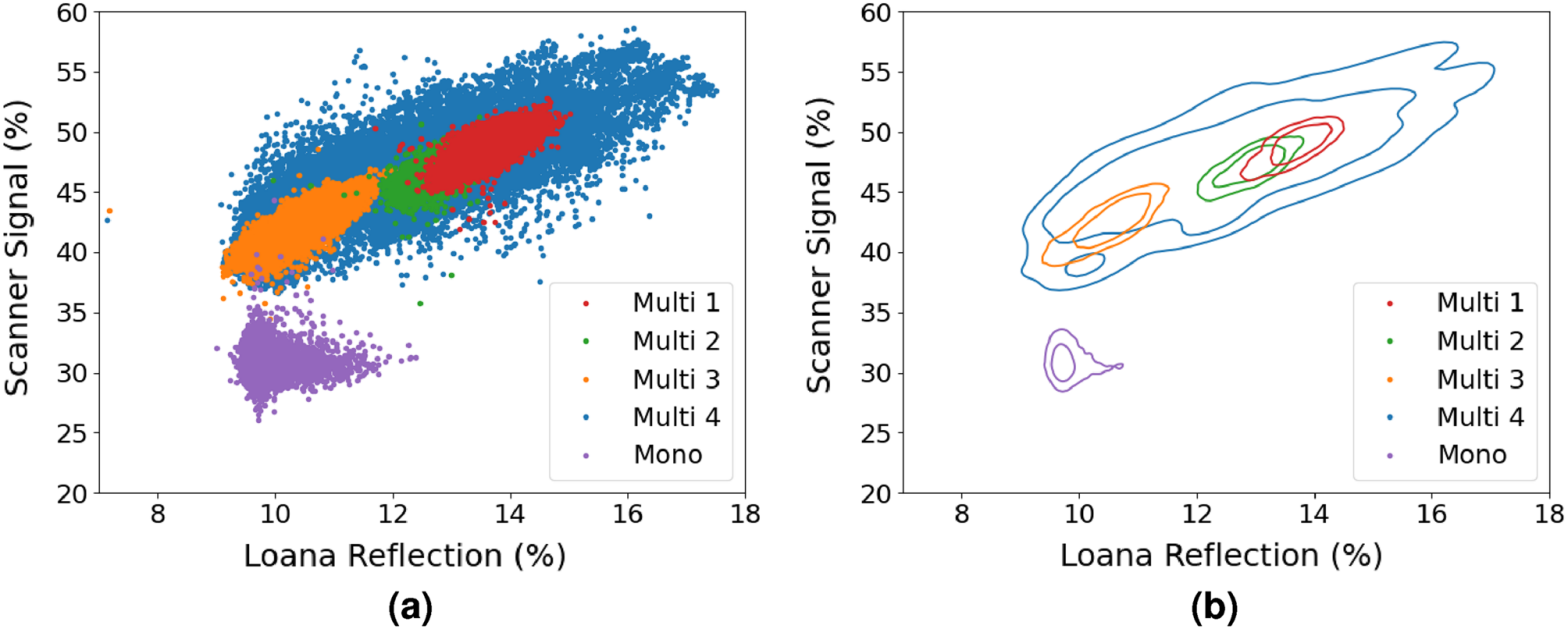
Comparison of the pixel intensity of the same region the wafers. (**a**) Raw data, (**b**) a statistical representation showing lines that represent 75% and 95% bounding lines.

The second metric used to quantify the similarity between the measurements is a direct comparison of pixel level data after scaling the images to a matching number of pixels and then aligning them using image registration. All of the raw pixel data from Fig. 3 is shown in Fig. 5a. Figure 5b uses contour lines to show a statistical visualisation of where most of the data points in Fig. 5a lie. The contour lines represent bounding lines that contain 75% and 90% of the data points for each data set. These bounding lines were determined using a kernel density estimate using Gaussian approximation to find the probability distribution. A good correlation is found between the measurements obtained for the scanner and the LOANA for the multi crystalline data, both within a sample and between samples. The mono-crystalline data did not fit the trend of the multi-crystalline data, nor did the variance between the two tools correlate.

For a quantitative statistical comparison of the data presented in Fig. 5 the Pearson’s correlation coefficient and the two sided p-value were determined for each wafer. The values determined are shown in Table 1 to two significant figures. The statistics were calculated using the python programming language with the pearsonr function from the scipy library. All the p-values are below 0.01, demonstrating a true statistical prediction of the Pearson coefficient. Note where a p-value of 0 is listed, this is the value returned by the function. The Pearson’s correlations found for the multi-crystalline data set were between 0.7 and 0.8, demonstrating a positive linear correlation. The mono-crystalline data was found to not have a linear correlation.

**Table 1:** A statistical analysis of the pixel level data after image registration between the intensity measure by the scanner and the LOANA.

Texture	Pearson correlation coefficient	Two sided p-value
Multi-1	0.70	0.0
Multi-2	0.71	0.0
Multi-3	0.79	0.0
Multi-4	0.75	0.0
Mono	− 0.037	4.2$\times$10$^{-8}$

## Discussion

Initial measurements were used to investigate if different scanner types had an impact on the measurement results. Solar precursors were scanned on both CIS and non-CIS flatbed scanners and showed significantly varying spatial results. The non-CIS scanner measured an increase in the signal towards the edge of the wafer along the imaging axis of the scanner. The CIS scanner did not measure this increase in signal. This increase in counts, or bow, only appeared along the imaging axis of the scanner, irrespective of the sample rotation. The bow depended on the sample location on the scanner, becoming larger when the wafer was closer to the edge of the scanner.

These results may be explained by the difference between the scanners’ collection optics. A CIS scanner, with each pixel having a magnification of 1 and each pixel having a dedicated lens, results in the same field of view for each pixel across the sensor. In contrast, the mirrors and lens within a non-CIS sensor create a changing field of view. This changing field of view across the sensor is referred to as vignetting and is corrected in a process referred to as a flat-field correction. With non-CIS scanners designed to scan paper and photos, their flat-field correction is designed to correct for the scattering profile of paper. As such, no bow is observed in data from either scanner if a photo or paper is scanned. This would suggest solar cells scatter more light than paper into the angles that the non-CIS scanner detects, as this would result in higher counts near the edges. Such a calibration process could be performed on specific solar cell texture’s however the calibration is unlikely to generalise over all textures that are currently used.

Measurements of several wafers with varying textures were then performed with the CIS based scanner, as it did not suffer from an incorrect flat-field correction, a property that may be sample dependent. The measurements were compared to reflection maps performed with a LOANA.

Several approaches have been used to qualify if a scanner can be used to monitor the absolute value and/or the uniformity of the reflection of textured wafers. A good correlation was observed for all metrics for the multi-crystalline wafers of varying textures. This is true both when comparing between samples and when comparing spatial data across a single multi-crystalline sample. This indicates that low-cost scanner technology is suitable for monitoring variations in the types of multi-crystalline texturing processes investigated here. However, these trends do not extend to the mono-crystalline wafer. The reflection of the random pyramid texture caused by the alkaline texturing process is not well captured by the scanner. This is believed to occur as a result of the different physical mechanisms of texture formation and how those textures scatter light. The metal assisted chemical texture, also known as black silicon, is a more statistical etching process (random process), with the resulting geometric shapes and reflection generally isotropic^9^. However, the alkaline texture used on the mono-crystalline wafers results in the formation of pyramidal shaped features. The variation in reflection comes from the pyramid coverage, and the ability for the light to be reflected off one pyramid and onto another. The resulting scattering profile has strong features relating to the geometry of the pyramids^10^.

Interestingly, there are also features that can only be observed within the original, higher resolution, scanned image. Specifically, there are repeating fine structures that run through the wafers. They run vertically in the first wafer, horizontally in the second wafer, vertically in the third wafer and fourth wafer, and horizontally in the fifth wafer which is the mono-crystalline wafer. These structures are remnants of the wire sawing used to cut the wafer from a block of silicon.

While it has not been confirmed that this pattern does not represent remaining damage from the sawing process, it is believed that it is more likely a remaining structure of the sawing process. The reasoning for this is that the texturing processes are not polishing steps, and so any larger surface variations, such as grooves from the wire saw, are expected to remain. Thus, with surface damage having been removed, some part of the sawing structure remained. This provides some motivation for the use of higher resolution optical inspection.

## Methods

**Figure 6:**
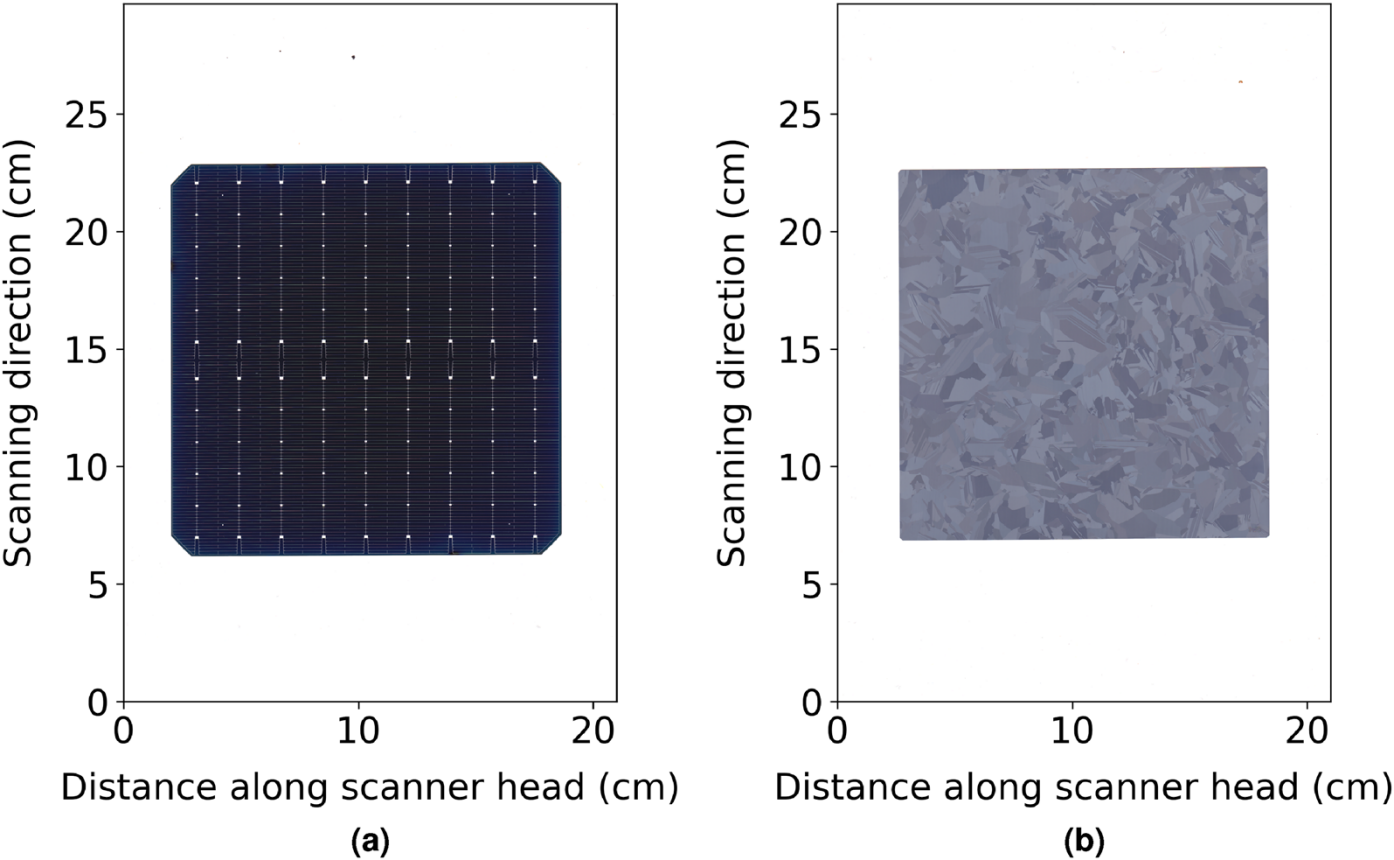
Example scan of (**a**) a silicon solar cell from a 2020 manufacturing line and (**b**) textured multi-crystalline wafer used in this study. The scan has 118 pixels per cm and was taken with a CIS based Canon Lide 300. The axes are labelled to represent the location of the scanner head and its movement.

A flatbed scanner consists of a line sensor that has its field of view sequentially moved across a region to build up a two dimensional image. They are intended for scanning documents and so typically cover a scan area of at least an A4 page, (210 mm $\times$ 297 mm), larger than the current and projected size of solar cells. An example of a scan of a solar cell from a commercial manufacturer is shown in Fig. 6. Both the fine features, being the metal fingers (<30 um), and the colour and uniformity of the cell can be observed. It is this low cost, high resolution, wavelength dependent information that this paper seeks to investigate for the application of monitoring solar cell texturing. As a scanner consists of line sensors that have two distinct axes. The first represents the axis to which the image sensor is aligned, and the second is the axis along which the image sensor’s field of view is moved, as indicated in Fig. 6. These axes will be referred to within this paper as the imaging axis and the axis of motion.

To determine if the scanner can provide a useful measure of reflectance of solar cell precursors’, a representative set of samples were acquired from large-scale solar cell manufacturers. The samples used were a set of p-type multi-crystalline wafers with an intentional variation of their texture. The textures were prepared using an AgNO$_3$ nano-pitting solution followed by an HF-HNO$_3$ etch to create sub-micron inverted features^8^. An increasing HF-HNO$_3$ etch time was applied from samples Multi-1 to Multi-4. Sample Multi-4 received an additional 30 second KOH (2 While several mono-crystalline wafers were measured, the most non-uniform mono-crystalline wafer is shown, to highlight the imaging sensitivity of the scanner.

To validate the results from the scanner, the wafer’s spatial reflection was also measured with a LOANA (pv-tools). The spatial reflection maps were extracted from a mapping routine at an illumination wavelength of 405 nm laser. The LOANA performed these measurements using an integrating sphere situated 4 mm above the wafer surface to measure the total hemispherical reflectance. It should be noted that the images acquired with the LOANA show three dark regions at the pixel values $x \in (20,80,140)$ and $y <10$. These regions represent the location of metal probes typically used to perform combined reflectance and quantum efficiency measurements for metallized solar cells. These regions are not related to the sample’s reflection and so are removed from the analysis. The wafer was mapped on a 1 mm grid taking a total of 20 min to provide a $155\times 155$ pixel image for the multi-crystalline wafers and a $160\times 160$ pixel image for the mono-crystalline wafers. The scanner took 10 s to provide a $5000 \times 5000$ pixel image, of which an $1800 \times 1800$ region contained the multi-crystalline wafer (pixel size of $\approx$ 80 $\upmu$m).

The image from the blue channel of the CIS scanner was compared to the image acquired with the 405 nm laser of the LOANA. As the values provided by the two tools report different metrics, being counts and hemispherical reflection, they can not have the same axis for their colour scale. To allow the variation in the different measurements to be shown on a comparable scale the colour scale was set to be between the 2nd and 98th percentile of the frequency distribution of the values for each image.

To allow direct comparison of pixel values, the image from the scanner was first down-sampled to the resolution of the LOANA, by taking the mean. The scanned image was then translated and rotated until its image features best aligned with the image from the LOANA. The pixel values were then compared both as raw data, but also using a Kernel density estimate. A Gaussian based kernel density estimate, using a bandwidth of 0.43, was used. As a kernel density estimate is a probability map of where the data falls, it enables the calculation of kernel values (or probabilities) for where data falls. The contour lines plotted in Fig. 5 represent constant values of the kernel density estimate, for which the integral of kernel density values larger than this contains 75% and 95% of the data points for each sample. In this way, these lines can be considered bounding lines that contain 75% and 95% of the data points for a single sample.

## Data Availability

All data generated or analyzed during this study are included in this published article and its supplementary information files.
